# Complete Genome Sequence of Bacillus subtilis Strain DKU_NT_02, Isolated from Traditional Korean Food Using Soybean (Chung-gook-jang) for High-Quality Poly-γ-Glutamic Acid Activity

**DOI:** 10.1128/genomeA.00525-18

**Published:** 2018-06-21

**Authors:** Man-Seok Bang, Hee-Won Jeong, Yea-jin Lee, Su Ji Lee, Sang-Cheol Lee, Jang-In Shin, Chung-Hun Oh

**Affiliations:** a Department of Medical Laser, Graduate School, Dankook University, Cheonan, Choongnam, Republic of Korea; b Department of Oral Physiology, College of Dentistry, Dankook University, Cheonan, Choongnam, Republic of Korea

## Abstract

The complete genome sequence of Bacillus subtilis strain DKU_NT_02, isolated from traditional Korean food using soybeans (chung-gook-jang), is presented here. This strain was chosen to help identify genetic factors with high-quality poly-γ-glutamic acid (γPGA) activity.

## GENOME ANNOUNCEMENT

Bacillus subtilis is a tuberous aerobic bacterium widely distributed in nature and used to manufacture various fermented soybean foods (1). Also, Bacillus subtilis changes, depending on the environmental conditions, to form spores (liposomes) and produce fermented material (2). This study was conducted to obtain strains for the production of high-quality fermented foods using Bacillus subtilis (3–5). As a result, we found Bacillus subtilis strain DKU_NT_02 to be useful in the production of high-efficiency fermented food.

Total genomic DNA was extracted using the Wizard genomic DNA purification kit (Promega, USA), according to the manufacturer’s instructions. Whole-genome sequencing of Bacillus subtilis strain DKU_NT_02 was performed using the Pacific Biosciences RS II sequencing platform at Macrogen (Seoul, Republic of Korea) (6).

A total of 1,027,289,253 PacBio raw reads were filtered, as per the read qualities. The cleaned reads were then assembled using RS Hierarchical Genome Assembly Process (HGAP) protocol version 3.0, as available in subread filtering from SMRT Portal 2.3, which generated 104,769 reads with about 202-fold depth genome coverage (7–9). The assembly resulted in a 4,014,255-bp genome sequence composed of one contig, with 43.6% G+C content.

The coding sequences were predicted by using Glimmer version 3.02. Functional annotation was achieved using the Prokka software. Functional categories were predicted using RAST version 2.0 (10, 11). A total of 4,148 coding sequences, 87 tRNAs, and 30 rRNAs were predicted. A plasmid was not found in this strain.

Access to whole-genome sequences for these strains will enable future investigations into the roles that the encoded metabolites might play in high-quality poly-γ-glutamic acid (γPGA) activity.

### Accession number(s).

The complete genome sequence Bacillus subtilis strain DKU_NT_02 was deposited in GenBank under the accession number CP022890.
